# Acute Pancreatitis in a Patient Taking Semaglutide

**DOI:** 10.7759/cureus.43773

**Published:** 2023-08-19

**Authors:** Femina Patel, Arnold Gan, Karen Chang, Kenneth J Vega

**Affiliations:** 1 Internal Medicine, University of California Riverside, Riverside, USA; 2 Internal Medicine, University of Southern California, Los Angeles, USA; 3 Gastroenterology and Hepatology, University of California Riverside School of Medicine, San Bernardino, USA; 4 Gastroenterology and Hepatology, Augusta University Medical College of Georgia, Augusta, USA

**Keywords:** post-cholecystectomy, abdominal pain, glucagon-like peptide-1 receptor agonist, semaglutide, acute pancreatitis

## Abstract

The Semaglutide and Cardiovascular Outcomes in Patients with Type 2 Diabetes (SUSTAIN-6) trial showed that semaglutide, a glucagon-like peptide-1 receptor agonist (GLP-1 RA), is effective in managing type 2 diabetes by stimulating insulin secretion and promoting weight loss. Though recent evidence suggests no increased risk of acute pancreatitis (AP) with subcutaneous semaglutide use, some studies report an increase in pancreatic inflammation with GLP-1 RAs. We present a case of AP in a patient recently started on subcutaneous semaglutide for type 2 diabetes. As GLP-1 RA use increases, clinicians should be aware of their potential to cause acute pancreatitis.

## Introduction

In the United States, the total cost of treating chronic diseases such as type 2 diabetes, hypertension, dyslipidemia, stroke, and coronary heart disease associated with obesity is approximately $1.72 trillion in a single year [1]. Glucagon-like peptide-1 receptor agonists (GLP-1 RAs) are antidiabetic medications that have successfully improved glycemic control in adults with type 2 diabetes mellitus (T2DM), facilitated weight loss, and reduced blood pressure [2-4]. Semaglutide, sold under the brand name Ozempic and Wegovy, is a popular GLP-1 RA that is administered subcutaneously once weekly. In June 2021, after being FDA-approved for weight reduction among patients with a body mass index of 27 or more with weight-related comorbidities, it is now more frequently prescribed by clinicians [4]. In addition, trials have shown semaglutide to be efficacious in reducing the rate of nonfatal myocardial infarction (MI), nonfatal cerebrovascular events, and cardiovascular death in T2DM patients [2]. 

Medication-induced acute pancreatitis (AP) is an adverse effect commonly associated with certain diuretics, anticonvulsants, and immunosuppressants. There have been cases of AP reported secondary to GLP-1RA use [5-10], but recent studies suggest there is no increased risk of AP with subcutaneous semaglutide use compared with non-users [11-13]. As semaglutide is the cornerstone in T2DM and weight management, careful risk/benefit consideration of drug continuation in patients with active or increased risk of AP should be performed. Here we present a case of AP in a diabetic patient who recently started on subcutaneous semaglutide for glycemic control.

## Case presentation

The patient is a 61-year-old female with a medical history significant for T2DM, hypertension, depression, and obesity (BMI 48.87). Long-standing outpatient medications consisted of metformin 500 mg twice daily, zolpidem 50 mg daily, lisinopril 10 mg daily, and atorvastatin 10 mg daily all by mouth as well as semaglutide 0.5 mg once weekly two months prior. 

**Figure 1:**
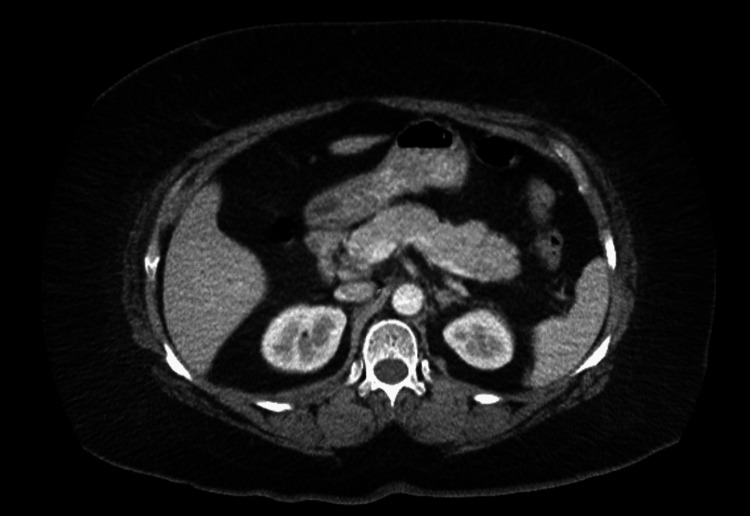
Computed tomography (CT) scan of the abdomen and pelvis with contrast showed no pancreatic pathology.

She presented to the emergency department with complaints of sudden onset abdominal pain that developed the previous day. Pain was described as severe and located in the right upper quadrant with radiation to her back. The patient denied any alcohol use, tobacco use, recreational drug use, or recent abdominal injury. Past surgical history was significant for cholecystectomy five years ago. All vitals were within normal limits, except for an elevated blood pressure of 178/80 mmHg. Complete blood count (CBC) and complete metabolic panel (CMP) were unremarkable, aside from elevated liver enzymes, aspartate aminotransferase (AST) 324 IU/L (normal range: 10-40 IU/L); alanine aminotransferase (ALT) 140 IU/L (normal range: 7-56 IU/L) and elevated lipase level (4,986; normal range: 10-140 U/L). Hepatitis serology was negative. Hemoglobin A1C was measured to be 5.9%. Computed tomography of the abdomen and pelvis with intravenous contrast revealed no acute abnormality (Figure 1). 

**Figure 2:**
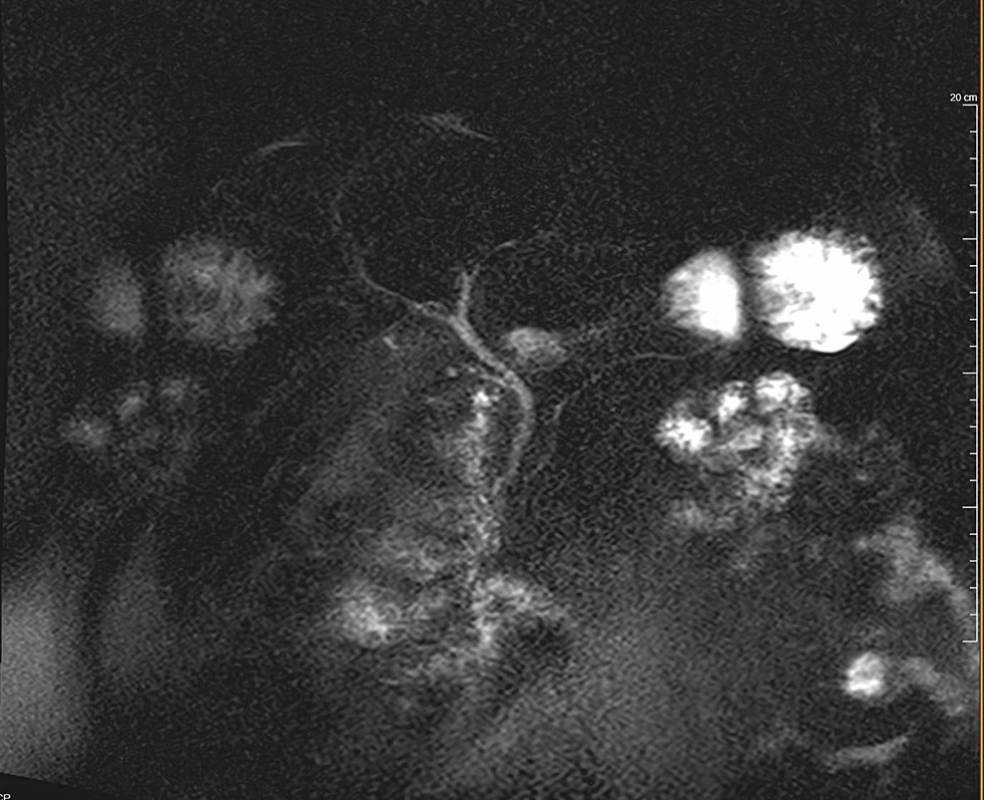
Magnetic resonance cholangiopancreatography (MRCP) showing a normal caliber common bile duct, pancreatic duct, and a remnant cystic duct status post cholecystectomy.

Due to characteristic abdominal pain and elevated lipase, she was diagnosed with acute pancreatitis. Magnetic resonance cholangiopancreatography (MRCP) was performed on hospital day 2 which did not show stones or any other findings suggestive of biliary tree pathology (Figure 2). 

The following day, the patient tolerated a solid diet and was discharged with the recommendation to not restart semaglutide. At her follow-up visit three months later, she remains without recurrence of abdominal pain. 

## Discussion

Subcutaneous semaglutide, a GLP-1 RA, is becoming more favored for glycemic control use among T2DM patients due to its additional weight loss properties, reduced risk of hypoglycemia, and once-weekly administration. These antidiabetic agents exert their therapeutic effects by potentiating glucose-dependent insulin secretion while simultaneously reducing glucagon release from pancreas [14], decreasing blood glucose levels. The direct stimulation of glucagon-like peptide 1 receptors located on pancreatic exocrine duct cells and pancreatic islet beta cells is hypothesized to cause acute or chronic pancreatitis by promoting hyperplasia, leading to increased pancreatic weight and exocrine duct occlusion [15-16].

There have been numerous reports linking GLP-1 RAs to the development of AP, chronic pancreatitis (CP), and pancreatic adenocarcinoma [5-10]. The Semaglutide and Cardiovascular Outcomes in Patients with Type 2 Diabetes (SUSTAIN-6) trial illustrated subcutaneous semaglutide was associated with AP at similar rates to placebo controls [2]. However, other studies have shown conflicting results [17]. Because AP associated with GLP-1 RAs might present atypically, the true prevalence may be higher due to underreporting. In addition, given the nature of observational studies, data could have been confounded since diabetic patients with indication for GLP-1 RA therapy often have concomitant risk factors for acute pancreatitis, such as obesity, longer diabetes duration, and being on other medications that independently increase pancreatitis risk. 

Our patient had a medical history significant for obesity and well-controlled T2DM, known risk factors for acute pancreatitis. Patients diagnosed with diabetes mellitus have a 74% increased risk of AP, whereas association with CP was found to be unclear [18]. This patient, who started weekly semaglutide 0.5 mg two months prior, did not report the typical history associated with AP, such as recent ethanol use, abdominal trauma, steroid use, viral infection, or autoimmune disease. Furthermore, laboratory workup was negative for hypercalcemia, hypertriglyceridemia, or leukocytosis. Imaging studies were also negative for biliary pathology. The combination of recent semaglutide use, lack of classic history findings of AP, remarkably elevated lipase levels, and negative imaging findings points towards subcutaneous semaglutide as the most likely perpetrator of this patient’s AP.

Although the SUSTAIN-6 trial illustrates that diabetic patients taking subcutaneous semaglutide are not at increased risk of developing acute pancreatitis, clinicians may need to consider discontinuation of this GLP-1 receptor agonist in patients who present with AP while taking this class of medication, particularly if they have other risk factors for acute pancreatitis.

## Conclusions

Clinicians should be suspicious of medication-associated AP in patients recently started on subcutaneous semaglutide, a GLP-1 RA, who present with AP along with unremarkable workup. Treatment of this condition consists of prompt discontinuation of subcutaneous semaglutide and initiation of therapeutic interventions for AP based on severity. Additionally, subcutaneous semaglutide should be cautiously prescribed in diabetic populations with increased risk factors for AP.
